# COVID-19 lessons learned: a global perspective

**DOI:** 10.1186/s13756-021-00992-x

**Published:** 2021-08-26

**Authors:** Kevin T. Kavanagh, Christine Pontus, Judith Pare, Lindsay E. Cormier

**Affiliations:** 1 Health Watch USA, P.O. Box 1403, Somerset, KY 42502 USA; 2 Massachusetts Nurses Association, Canton, MA USA; 3 College of Nursing and Health Sciences, University of Massachusetts Boston, Boston, MA USA; 4 Department of Biological Sciences, Eastern Kentucky University, Lexington, KY USA

## Abstract

One June 15, 2021, infectious disease authorities from around the world participated in a joint webinar to share experiences and lessons learned in combatting the COVID-19 pandemic. One of the overriding goals of the conference “*COVID-19 Lessons Learned: A Global Perspective”* was to provide documentation of worldwide COVID-19 response strategies, in order to combat the plethora of misinformation and conspiracy theories that are being actively disseminated. This misinformation is having a profound negative impact on controlling the pandemic in many countries. Misinformation which was addressed in the conference included challenging the seriousness of COVID-19 infections, a refusal to recognize aerosolization as the major mechanism of spread, a belief that schools can be opened safely without implementation of extensive control strategies, and that masks and vaccines are not effective. A second goal was the identification of common strategies between nations.  Common strategies included the implementation of a range of closures, mask mandates, travel bans and the need for expanded testing. But of utmost importance there was recognition of the need to implement a coordinated national strategy, which is depoliticized and led by scientists.

## Introduction

Infectious disease authorities from around the world presented and participated in a joint webinar on 15 June 2021 to share experiences and lessons learned in combatting the COVID-19 pandemic—See Table 1. It is hoped that a worldwide perspective will aid in adoption of effective strategies and to mitigate the epidemic of misinformation which is often predicated on local or regional mistrust of government and politics.

**Table 1:** Speakers and countries represented at *COVID-19 lessons learned: a global perspective*

Imogen Mitchell	Australia
Sebastian Hoehl	Germany
Vineeta Gupta	India
Paul Yonga	Kenya
Matthias Maiwald	Singapore
Jesús Rodríguez-Baño	Spain
Stephanie Dancer	United Kingdom
Kevin Kavanagh	United States
Carolyn Clancy	United States Dept. of Veterans Affairs
Beth Taylor	United States Dept. of Veterans Affairs

## Misinformation constraining pandemic responses

One of the overriding goals of the conference “*COVID-19 Lessons Learned: A Global Perspective”* was to provide documentation of worldwide strategies to combat the plethora of misinformation and conspiracy theories which are being actively disseminated. In many countries, this misinformation is having a profound negative impact on controlling the pandemic.

In Brazil, misinformation has been reported to be spread by politicians and preachers. This has allegedly resulted in indigenous populations greeting public health officials with bows and arrows [1]. In India, misinformation encouraged the public to search for ineffective treatments and homeopathic medications not backed by evidence demonstrating efficacy in COVID-19 [2]. In Europe, a European Union document described a Russian disinformation campaign pushing fake news, making it harder for the Union itself to implement its pandemic response [3]. More recently, the United States identified and denounced a Russian disinformation campaign shedding doubt over the effectiveness of vaccines [4].

Disinformation was often fanned by political rallies where public health advice was ignored and community leaders set an example of not following COVID-19 containment strategies, impeding their acceptance by the general population. Such rallies were seen in many countries, including the United States, India, Brazil, Kenya and Spain. As evidenced in Spain and the United States, the focus of the rallies was the call of freedom. As pointed out, “infringing on freedom has become the main point of attack on restrictions and regulations during the Covid-19 pandemic, but freedom is more than simply being able to do what we want” [5]. Of course, freedom from public health strategies will too often usher in death or disability from Long Covid.

Some political rallies were held indoors. Others were mass outdoor gatherings. In the case of the United States, pictures from a recent Independence Day celebration showed tightly packed maskless individuals, including presumably unvaccinated young children. These events demonstrate that many, including political and other leaders, are not taking the pandemic seriously [6].

Baker et al. classified a country’s COVID-19 response strategies into five categories: [7] Exclusion Strategy (some Pacific Islands), Elimination Strategy (Singapore and Australia), Suppression Strategy (European Union and United Kingdom), Mitigation strategy (USA, Brazil, and India), and No Substantive Strategy.  The success of any chosen strategy generally depended upon the degree and uniformity of support the public held for preventative strategies. However, misinformation impedes this support.

A mitigation strategy was adopted in large areas of the United States, Brazil and India. In this strategy, attempts were made to try to flatten the case curve until herd immunity was reached or a vaccine became available. The overarching goal, which was not always achieved, was to prevent the healthcare system from being overrun with COVID-19 patients.

At the other end of the spectrum is an elimination strategy as adopted by Australia and Singapore. In this strategy, there is a zero tolerance for the SARS-CoV-2 virus. In Australia, cities were closed even if there were only 1 or 2 cases. In both countries, everyone entering the country were required to quarantine. In a few countries the Delta Variant prompted the quarantine time period to be extended to 21 days [8]. With the Delta Variant causing mild and asymptomatic infections in the those vaccinated [9], vaccination does not exempt one from compliance with advisements. For example, in Singapore, starting after a case surge in May 2021, all hospital staff were tested for COVID-19 every two weeks, and all newly admitted patients were tested. Both Australia and Singapore have implemented the use of cell phone apps for tracking of residents to facilitate case identification [10–12].

Many conspiracy theories are rooted in a nation’s culture, economics, or politics. However, it becomes more difficult for these theories to remain viable and attack national or regional advisements when the strategies being enacted are similar to those enacted by other countries around the globe.

## Common misinformation talking points which have inhibited pandemic responses

### Misinformation: SARS-CoV-2 is the flu

This conspiracy theory stems from the fact that seasonal influenza disappeared at about the same time COVID-19 emerged. The premise is that SARS-CoV-2 is actually just severe cases of the seasonal flu which are misdiagnosed. This argument may seem plausible in countries such as the United States, where COVID-19 cases surged and influenza disappeared, but the theory can easily be disproven by looking at data from Singapore. As described by Dr. Matthias Maiwald [13], Singapore instituted a stringent elimination strategy, resulting in both the less infectious influenza virus and the highly infectious SARS-CoV-2 virus to almost totally disappear. All respiratory viruses had a substantial decrease during Singapore’s lockdown period. Relaxation of strategies resulted in the reappearance of some viruses. Approximately 13 weeks after reopening, rhinovirus and enteroviruses reappeared. Later in 2020, adenoviruses reappeared but influenza remained at almost non-existent levels throughout the remainder of the year [14].

### Misinformation: fatality rates of SARS-CoV-2 are overestimated

There is actually a denial of and underestimation of true deaths rather than an overestimation. For example, in early June of 2021, the official death toll In India was just over 350,000, but may actually have been as high as 4.2 million individuals [15]. Many strategies can be enacted to lower actual numbers, such as requiring a COVID-19 test in a country with a fragmented and underdeveloped healthcare system, or not counting patients with almost any co-morbidity for a virus which preys on the elderly. The latter strategy can reduce deaths by 50% to 80%. The same was true in the United States where the number of counted cases was approximately 600,000 but actual deaths using excess mortality data was estimated by the Institute for Health Metrics and Evaluation to be over 950,000 [16].

### Misinformation: herd immunity can be achieved without protective measures

Despite lack of any firm evidence for pandemic subsidence, herd immunity was advocated as an effective strategy for poor countries [17] and was declared to have been reached by the Fall of 2020 [18–20]. However, a crushing wave of COVID-19 affected the world over the winter holidays, followed in many countries by another wave caused by the Delta Variant.

The quest for herd immunity with or without a vaccine has been made almost non-achievable with the emergence of the Delta Variant where the R0 is estimated to be greater than twice that of the wild type of virus. This means that greater than 85**%** of the population would have to be immune before herd immunity is achieved. In addition, SARS-CoV-2 is actively infecting the animal population particularly cats, dogs, and minks. Zoos are even starting to implement vaccination programs for large cats, apes, bears, and ferrets [21].

### Misinformation: we are overly concerned regarding children and the opening of schools

The excuse often given in many regions of the United States is that other countries have shown that schools can be opened safely, and children are at low risk for the disease.  However, what is left out is that extensive strategies to identify carriers and prevent spread were enacted to allow the students to attend schools in as safe of an environment as possible. Most countries recognize schools as an important source of SARS-CoV-2 spread. School closures have been implemented in many countries, including the United States, United Kingdom, Germany, Kenya, and Singapore.

In Germany, there were school closures. When schools were reopened, there were mask mandates, social distancing and rapid antigen testing twice weekly for all students and teachers. A positive test was then confirmed with PCR testing. Even with these interventions, there were at least 86 outbreaks documented in daycare nurseries and schools.

Dr. Sebastian Hoehl presented data from the Robert Koch Institute which found that during Germany’s third wave, there was a shift in the age distribution of cases to younger individuals. Patients between the ages of 5 and 19 years old frequently became infected with SARS-CoV-2 [22].

In the United States, the CDC recommends a number of strategies which schools “might specifically implement”, such as social distancing, use of masks and screening for COVID-19 [23]. On 19 Mar. 2021, the CDC decreased the social distancing in schools from 6 to 3 feet [24]. A recommendation which appeared to be based upon droplet spread and lacked detailed recommendations regarding aerosolization of the virus.

On 7 May 2021, the CDC recognized that aerosols are a major mechanism of SARS-CoV-2 spread [25]. However, too few school districts have upgraded their ventilation systems. Currently, the CDC recommends “increased” and “improved ventilation” of schools but lacks specifics for implementation [23].

Many school districts have not implemented mandatory systematic testing of all students and teachers. CDC recommendations are to “offer” not “mandate” regular testing in schools. Cited barriers include “privacy concerns, operational complexity, and financial concerns” [23].

For both masks and testing interventions, the recommendations vary depending upon vaccination status, a distinction with questionable validity with the emergence of the Delta Variant and the reports of vaccine breakthrough infections.

### Misinformation: three to six feet away and you are safe

There has been a denial by many governments regarding the airborne transmission potential for SARS-CoV-2. This, in part, emanated from original guidelines produced by the World Health Organisation (WHO). Dr. Stephanie Dancer, NHS Lanarkshire, Scotland, aptly asserted that aerosolization is a major mechanism of spread for SARS-C0V-2 and proper ventilation in buildings is of utmost importance [26]. Viral particles and small droplets can aerosolize with breathing and speech, and float in the air, particularly in poorly ventilated environments [27]. Viable SARS-CoV-2 have been cultured in the air 3 h after aerosolization. It should be noted that two other highly infectious airborne pathogens, tuberculosis and measles have never been successfully cultured in the air. The United States has been exceedingly slow to enact strategies regarding aerosolization and took until early May in 2021 to recognize that aerosolization was a major factor of spread of SARS-CoV-2.

### Misinformation: masks do not work

This misinformation is based upon the fact that the SARS-CoV-2 virus is less than 1 micron in size and thus would not be effectively filtered using even an N-95 mask. This argument asserts the ineffectiveness of mask recommendations but ignores the evidence that the transmitted particle size is most often much larger, in the form of droplets. 

All of the countries represented at the conference have mask mandates as part of their strategy, including Kenya, Spain, Germany, Australia, Singapore, United Kingdom, and the United States. Dr. Paul Yonga described how Kenya immediately implemented strategies to control the spread of SARS-CoV-2. The country of Kenya even mandated masks two weeks before their use was recommended by the WHO [28].

Dr. Stephanie Dancer explained that there is a continuum of viral droplet particle sizes which are produced by SARS-CoV-2 [26]. Those under 100 microns can form aerosols. These particles can be effectively filtered with N-95 masks but also well-constructed and well-fitted cloth masks. Because of concerns of aerosolization, Germany mandated the use of surgical or N-95 masks in most “shops and public transport” [29]. Singapore mandated the public to wear at least surgical or cloth masks since mid-April 2020 and had a pre-pandemic stockpile of approximately three N-95 masks for every resident [13, 30].

Universal use of masks are of utmost importance, since as pointed out by Dr. Dancer, asymptomatic spread may account for over half of SARS-CoV-2 transmissions [26].

### Misinformation: antivaxxers

The antivaxxer movement tends to downplay the seriousness of SARS-CoV-2 by using many of the above misinformation strategies and to exaggerate the exceedingly rare vaccine complications which have been reported. Similar with most of the European Union and the United States, the antivaxxer movement was a major concern in all countries, except Spain. Dr. Jesús Rodríguez-Baño reported that greater than 75% of Spain’s population were confident in the coronavirus vaccine and additional percentages would become vaccinated anyway. In Kenya, there is a critical vaccine shortage and supply does not even begin to meet demand.

## Overriding lesson learned—need for a comprehensive, coordinated national and world response

The second overriding message was the importance of having a coordinated national strategy and healthcare system. Several of the countries, including the United States, Spain and Australia are federated where governmental power is concentrated in Regions and States. In the United States, pandemic response was largely directed by the States, with the Federal Government assuming an advisory role. In Spain, emergency powers initially placed pandemic governance at the federal level, but with later surges this shifted to regional decision making. In Australia, the country had an overarching Australian Health Principle Protection Committee, composed of the federal and jurisdictional chief health officers to oversee and guide the emergency pandemic response. The UK formulated SAGE, an independent committee of scientists to advise the government on pandemic control strategies.

Social disparities must also be addressed in a comprehensive pandemic response. It is not only a moral imperative, but those with a lower social economic status are particularly vulnerable to infections by the virus. Low-income migrant workers in Singapore became the subjects of a fourth wave which hit the country. In Germany, a major outbreak was associated with a meat processing plant. As stated by Dr. Sebastian Hoehl, “This was one of the first times that we saw that those that are the most affected by this cluster, this outbreak, were very low paid workers.” [22]. During the winter holiday surge, Germany also recorded a “much higher” number of cases in lower income communities. In Kenya, as in most low-income countries, vaccines are lacking which not only places their citizens at greater risk for COVID-19 but also creates a basis for the emergence of new variants. As stated by Dr. Jesús Rodríguez-Baño “It is a global failure, is the way the vaccines have been distributed across the world. Once again, those of us who live in developed countries are lucky enough to have much more probabilities of receiving the vaccines earlier than those living in low-income countries ….” [31].

Protecting hospital staff and patients is of utmost importance. NHS Lanarkshire in Scotland started to vaccinate their staff against SARS-CoV-2 in early December 2020. The virus entered all three acute care hospitals “from the community via staff both asymptomatic and pre-symptomatic.” Transmission then occurred between staff and patients, and from patients on one ward, and then to other wards [26]. Naturally ventilated wards contributed towards spread since it was winter, and staff were reluctant to open windows. In Singapore, after case clusters occurred in hospitals, all staff were tested every two weeks, and if unvaccinated, every week. Pooled testing was used to facilitate implementation of this strategy. In the United States, healthcare-acquired infections are effectively not tracked. The metric used in the United States for hospital onset SARS-CoV-2 infections, does not require reporting unless the patient is currently hospitalized and the infection occurred 14 or more days after admission. In addition, patients “should not be counted” if they become asymptomatic and are removed from isolation precautions [32]. The average length of hospital stay is 4.6 days [33]. Hence, a hospitalized patient who left the hospital at day 13 would not be counted as a Hospital Acquired Infection (HAI). This metric does not provide an adequate method for accurate accounting of HAI.

All front-line workers should be provided with adequate PPE, including N-95 masks, and with a robust testing infrastructure. Increases in testing capacity for COVID-19 screening in the workplace and schools is needed. Pool testing can be used to increase testing efficiency and conserve resources.

For example, Germany mandated testing of all students, teachers and industrial workers twice a week, and testing is recommended in all workplaces. Rapid antigen tests are used. They are relatively accurate during the time period of symptom onset, when viral load is high. In Australia, it became apparent that staff in designated quarantine hotels could become vectors for the disease and as a result, their movements are restricted, and they are tested daily.

Travel restrictions and bans are important strategies which have been implemented by most nations. They are intended to limit the spread of variants, delaying entrance into a country and hopefully allowing effective case tracking for the few cases which emerge. SARS-CoV-2 entered Germany on 17 January 2020, from an air traveler who flew into the country from China. Dr. Sebastian Hoehl presented extensive data regarding the ineffectiveness of symptom and temperature screening and stressed the impact that asymptomatic carriage has on spread of the virus. Dr. Imogen Mitchell from Australia emphasized the importance of controlling viral spread. Australia banned cruise lines and advised against citizens traveling overseas. Everyone entering the country would have to be quarantined for 14 days, usually at designated hotel quarantine facilities. During outbreaks, Australia also closed borders and travel between states and territories to limit the spread of SARS-CoV-2. Singapore, a city-state, performed similar bans and quarantines for persons entering the country.

Of all of the industrialized nations, the United States appeared to have one of the most substantive deficits in how the public healthcare system was able to address the pandemic, and the country lacked a national coordinated response. To help alleviate this deficiency, Acting Deputy Secretary Carolyn Clancy and Undersecretary Beth Taylor described how the United States Department of Veterans Affairs (VA) activated its Fourth Mission. The Fourth Mission does not solely aid veterans, it is designed to aid the country in healthcare emergencies, including pandemics. During the COVID-19 pandemic, the VA hospitalized over 400 non-veterans at VA facilities and outsourced over 1000 healthcare staff to community nursing homes and non-VA facilities. With the VA having the largest infrastructure of any healthcare system in the United States, many at the conference felt that the VA could possibly serve as an infrastructure for development of a national public healthcare system, along with coordinating and delivering a unified national response for the next pandemic.

A robust system of genomic testing for variants should be implemented in all nations. This includes the testing of a substantial portion of randomly selected positive specimens, in order to enable effective tracking of community transmission.

But above all, we must start to pay attention to respiratory pathogens the same way we do for food, surface and waterborne pathogens [34]. We must recognize the need and require upgraded indoor ventilation systems, air filtration and sanitization along with implementing stringent strategies to halt the transmission of SARS-CoV-2. This will not be an easy task, as observed by Dr. Matthias Maiwald [14]. It was relatively easy for public health strategies to cause the disappearance of almost all respiratory viruses, including seasonal influenza. By way of logical extension, it may be concluded that SARS-CoV-2 must be extremely effective in transmission to be so persistent around the world.

*Videos and slides of the conference presentations, along with provisions for obtaining AMA Category I continuing education credits for physicians are available free of charge at *http://healthconference.org.

## Data Availability

All presentations can be viewed at https://healthconference.org.

## Abbreviations

AMA: American Medical Association
CDC: Centers for Disease Control and Prevention
NHS: National Health Service
PPE: Personal Protective Equipment
SAGE: Scientific Advisory Group for Emergencies
UK: United Kingdom
USA: United States of America
VA: United States Department of Veterans Affairs.
WHO: World Health Organisation
